# The National Insurance Scheme and the Supply of Drugs

**Published:** 1911-07-22

**Authors:** 


### THE NATIONAL INSURANCE SCHEME AND THE SUPPLY OF DRUGS.

The opinion is growing on both sides of the House of Commons that it would be unwise to hurry the National Insurance Bill through Parliament, and pressure will be brought to bear on the Government to alter the original intention of passing the measure this session. Possibly the debate on the clauses in Committee will make clear many points that are at present somewhat obscure, one of which is the question of the supply of medicines to insurance patients. With regard to this matter much doubt prevails, and all that is definitely known is that the dispensing of medicines is not to be done by medical practitioners except in special circumstances. Friendly societies and Local Health Committees are to make provision for the supply of proper and sufficient drugs and medicines to insured persons, but with whom and on what terms the arrangements are to be made is not indicated in the Bill. In his speech in the House of Commons, in which he explained the scheme, the Chancellor of the Exchequer gave it to be understood that the dispensing was to be done by chemists; but the Bill does not provide for this, and officials of friendly societies have expressed the intention of establishing a drug warehouse and branch dispensaries, in which case they would be independent of pharmacists already established in business. If this intention were carried out it would entail a loss of millions of customers to chemists, which would be a very serious matter indeed. Pharmacists are hoping that some amendment will be made which would prevent this loss, and that, in regard to the supply of drugs, the German method will be adopted. In Germany there is a fixed schedule of prices for medicines, and an insurance patient may take his prescription to any pharmacy, where it will be dispensed at the official rates. At present, however, Mr. Lloyd George is in favour of a capitation fee, but this would be extremely difficult to fix. He calculates that a sum of £4,200,000 will permit the average payment of a capitation fee estimated at 6s. per head for each person insured "for medical attendance and the drugs, dressings and appliances used in domiciliary treatment." Until the scheme has been working for a year or two it will be impossible to estimate the cost of the necessary drugs and appliances, unless the German figures are taken as a basis. In Germany the cost of a year's supply of drugs for something like twelve million insured persons is about half the whole amount Mr. Lloyd George considers sufficient to pay for medical attendance and drugs; so it is obvious the Chancellor has not based his estimate upon German experience.

If the term "proper and sufficient drugs and medicines" is to include such things as bandages, hot-water bottles, and other invalid requisites it would be a hazardous speculation to fix a capitation fee, and nothing but an official schedule of prices would meet the case in a satisfactory way. The principle of differentiating between the medical man as the prescriber and the pharmacist as the dispenser is welcomed by pharmacists, but until their position is more clearly defined they cannot but regard the proposed scheme with considerable anxiety.

